# HPV vaccination programs have not been shown to be cost-effective in countries with comprehensive Pap screening and surgery

**DOI:** 10.1186/1750-9378-8-21

**Published:** 2013-06-12

**Authors:** Judy Wilyman

**Affiliations:** 1 Media and Communication, University of Wollongong (UOW) School of Social Sciences, Wollongong, Australia

**Keywords:** Cervical cancer, Human papillomavirus virus (HPV), Genotype, Infectious diseases, Gardasil®, Public health policy, Vaccination programs

## Abstract

Pap screening combined with loop electrosurgical excision procedures (LEEP) is almost 100% effective in preventing cervical cancer mortality yet many countries with these procedures have now implemented broad HPV vaccination programs. HPV vaccines have not been demonstrated to be more effective or safer than Pap screening in the prevention of cervical cancer and Pap screening will still be required even in vaccinated women. The HPV vaccine costs Au$450 per person and it does not protect against ~30% of cancer. This investigation analyses the cost-effectiveness of using the HPV vaccine in countries where Pap screening and surgical procedures have already reduced cervical cancer mortality to very low rates. Cost-effectiveness of vaccination programs is being determined by mathematical models which are founded on many assumptions. It is necessary to examine the rigor of these assumptions to be certain of the health benefits that are predicted. In 2002 scientists concluded that HPV 16 and 18 were the central and independent cause of most cervical cancer. This conclusion was based on molecular technology. If HPV 16 and 18 infections are the central and independent cause of most cervical cancer then the incidence of HPV 16 and 18 should vary with the incidence and mortality of cervical cancer worldwide. This correlation does not exist. It is also observed that the majority of HPV 16/18 infections do not lead to cervical cancer. This indicates that other etiological or ‘risk’ factors are necessary for persistent HPV infection to progress to cancer. The benefits of HPV vaccines have been determined by using pre-cancerous lesions in young women as a surrogate for cervical cancer. This surrogate is found to be inadequate as an end-point for cervical cancer. Clinical trials have only provided speculative benefits for the efficacy of HPV vaccines against cancer and the long-term risks of the vaccine have not been established. Pap screening will still be required in vaccinated women hence HPV vaccination programs are not cost-effective, and may do more harm than good, in countries where regular Pap screening and surgery has already reduced the burden of this disease.

## Background

Knowledge of the etiology of cervical cancer has been developed over the last century and during this time many lifestyle and environmental factors have been implicated in the etiology of this disease [1,2]. In 2002 it was stated that Human Papillomavirus (HPV) genotypes 16 and 18 are responsible for causing approximately 70% of cervical cancer worldwide [3]. This claim was made in 1995 based on the use of new hybridization technology for detecting HPV DNA that was stated to be ‘truly sensitive and specific’ [4]. Prior to 1995 the detection of HPV DNA in different tissues was known to be unreliable and the sensitivity of the results varied with the different techniques [5]. A causal theory based only on the presence of HPV genotypes is strongly dependent upon the accuracy and precision of the biotechnology used for detection [6]. Identification of HPV genotypes in the anogenital tract is also complicated by the fact that there are at least 40 HPV types present making it difficult to distinguish the causal factors for cervical cancer [6].

In 1995, Bosch et al. set out to characterise the global distribution of HPV genotypes because they knew this was ‘essential to the development of vaccination strategies to curb the burden of cervical cancer’ [7] p.797. In this study of 1000 cervical cancer tumors it was found that 93% contained HPV DNA [7]. This international study used new polymerase chain reaction-based (PCR) assays to detect more than 25 HPV types in 1000 specimens. In 1999, the 7% of tumors that were originally found to be HPV negative in the Bosch et al. study were re-analyzed using different techniques and assumptions [8]. After re-analysis Walboomers et al. claimed that 99.7% of tumors contained HPV DNA [8]. This evidence and other case–control studies led scientists to claim that persistent infection with HPV 16 and 18 is the main and determining factor in the etiology of most cervical cancer [3,8]. Consequently it was considered that a vaccine might be beneficial in reducing the global burden of cervical cancer [3]. This conclusion was based solely on the accuracy of the detection methods and assumptions that were used to attribute causality to HPV genotypes 16 and 18.

Whilst PCR methods are more sensitive and specific than liquid hybridization techniques and enable the identification of different genotypes, the specificity of this technique depends upon the type of primer used: type-specific or broad-spectrum [6]. The Bosch et al. 1995 study used the broad-spectrum MY11/09 method to genotype HPV-DNA [7]. The nascent technology used in this study was only available from the mid-nineties so the evidence for the causality of different HPV genotypes was based on a small number of studies between 1995 and 2002. Prior to 2002 a multi-factorial etiology was believed likely with HPV being a necessary factor but not a sufficient cause [2].

By 2002 scientists were proposing that human papillomavirus (HPV) Type 16 and 18 was the ‘first ever identified necessary cause of human cancer’ [3]. This suggests that cervical cancer does not and will not develop in the absence of HPV DNA [3]. The claim hasn’t been sustained because some investigators observe that HPV infection cannot be found in every patient with cervical cancer [2]. Scientists have found that persistent infection with one of 15 genotypes of HPV can lead to cervical cancer and it is stated that HPV genotypes 16 and 18 are the cause of the majority of cervical cancer worldwide [3,8]. The International Agency for Research on Cancer (IARC) working group also acknowledged in 2005 that there are cofactors that are associated with HPV infection and cancer development [9]. In other words, an HPV infection does not progress to cancer without the co-factors being present.

Several risk factors have been identified including infection with other sexually transmitted infections (STI’s), high parity, smoking and hormonal contraceptives [9]. The strength of these risks (co-factors) is variable and even though it was known in 2002 that co-factors were required for pathogenesis it was still believed that a vaccine targeting HPV 16 and 18 would prove effective [9].

Clinical trials to test the hypothesis that the quadrivalent HPV vaccine would be effective against cervical cancer, and not just the prevention of HPV 16/18 infection, were started in phase 3 trials in 2003 [10,11]. These trials investigated pre-cancerous lesions in 12,167 women (15–26 years old) and were completed in 2007 [10]. Yet the quadrivalent HPV vaccine was approved for the European and US market in 2006 [12,13]. The HPV vaccine, Gardasil® was developed and marketed to women as an effective prevention for cervical cancer after only 4 years of testing for efficacy against pre-cancerous lesions [11,14].

This paper examines the epidemiology of HPV infection and its progression to cervical cancer in different countries. It analyses the assumptions that have been made to claim that a vaccine against cervical cancer is cost-effective (CE) in countries with already established Pap screening programs. The cost-effectiveness of HPV vaccines has been determined using mathematical models that are limited by the assumptions they are based on [15]. An independent assessment of these assumptions is essential to population health and the effective distribution of health resources to the community. This paper provides an independent assessment of these assumptions and re-evaluates the cost effectiveness of broad vaccination programs that have been implemented in many countries.

## Review

### The global distribution of HPV 16 and 18 and cervical cancer incidence and mortality

Infection with HPV 16 and 18 has been stated to be the central and independent cause of cervical cancer. This infers that no other factors are required for pathogenesis to occur. However, epidemiologists observe that an infectious agent is an insufficient cause of disease [16]. This is because pathogenesis of any infectious agent is dependent upon environmental and lifestyle characteristics [16]. If HPV 16/18 are the central and independent cause of cervical cancer then the incidence of cervical cancer mortality would vary with the incidence of HPV 16 and 18 globally. This correlation does not exist. Cervical cancer is significantly higher in the developing countries than developed countries even though HPV 16/18 infections are not higher in these countries.

In 1995 it was observed that the distribution of HPV genotypes 16 and 18 was similar among all countries: developing and developed [7]. HPV 16 is identified as the dominant sub-type (62%) in squamous cell carcinoma (SCC) and cervical adenocarcinoma (CAC) in all countries and HPV 18 has a global frequency of 8% [11,17]. Yet cervical cancer rates vary significantly between countries. There are very high rates of disease in the developing countries and very low rates in developed countries [18]. This contrast is also observed between the Australian indigenous and non-indigenous populations. The indigenous population has twice the incidence of cervical cancer and five times the mortality rate [19]. This illustrates the influence of environmental and lifestyle factors in the pathogenesis of HPV infections [19].

In the 60’s and 70’s many developed nations had the same high rates of cervical cancer as the developing nations today but mortality was reduced due to changes in environmental and lifestyle factors and the introduction of Pap screening programs [18]. China also had a high incidence of cervical cancer in 1985 but this was reduced from 17.8 to 6.8/100,000 women by changes to risk factors by 2002 [18]. In 2003 it was observed that HPV 16 was slightly *less prevalent* in the countries with the highest rates of cervical cancer [17]. This led to the suggestion that a vaccine targeting HPV strains 16 and 18 may prevent more invasive cervical cancer in developed nations where cervical cancer rates are low, than in the developing countries which carry the highest burden of this disease [17].

Bosch et al. state ‘HPV 16 has been found to be the most prevalent HPV type in cytologically normal women as well as women with cervical intraepithelial neoplasia (CIN) and women with cervical cancer’ [7] p797. It is known that HPV infections are mostly self-limiting and harmless [13] p.3 and the global distribution indicates that the ‘risk’ of infection from HPV 16 and 18 is similar in all countries but the risk of ‘disease’ (cervical cancer) is higher in lower socioeconomic countries and communities.

Despite the lack of correlation between HPV 16 and 18 with the burden of cervical cancer globally, it was claimed that infection with high-risk HPV is the central etiological factor in cervical cancer worldwide and independent of other risk factors [7] p796. Further evidence that HPV 16 and 18 are not independent factors in the development of cervical cancer can be observed by examining the lifetime risk of this disease in different countries. Studies have shown that the incidence of HPV infection in women worldwide is approximately 80% but the lifetime risk of developing cervical cancer before the age of 64 is only 0.8% in a developed nation [18]. This risk increases to 1.5% in developing countries [18]. This difference cannot be fully explained by the presence of screening programs in developed countries because it is known that cancer is an uncommon outcome of all high-grade lesions in women [20].

**Figure 1:**
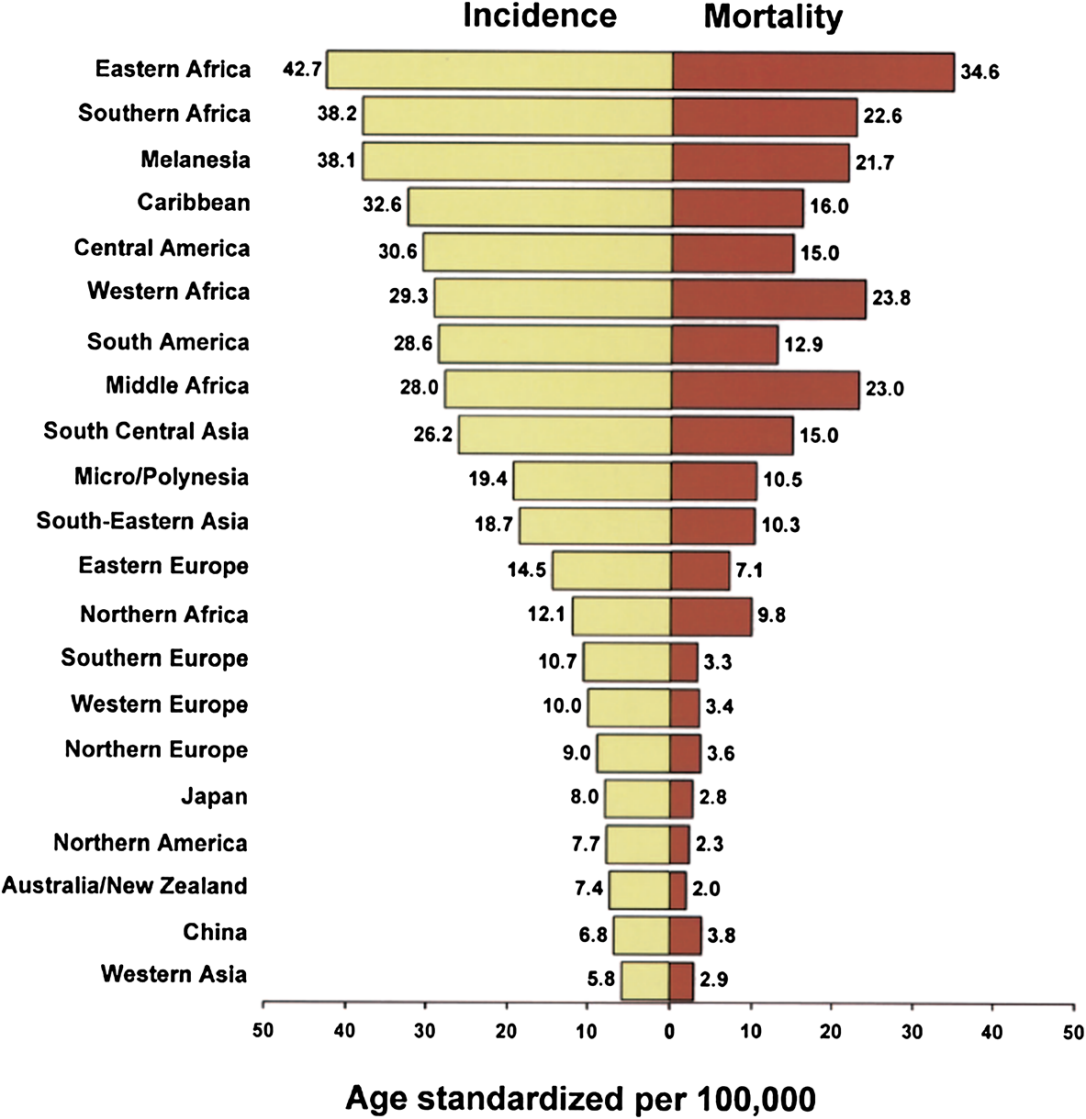
**Age-standardised incidence and mortality rates for cervix uteri cancer worldwide **[18]**.**

*If* HPV 16 and 18 are the central independent cause of most cervical cancer then the incidence of these subtypes should be higher in the developing nations where the incidence and mortality of cervical cancer is the highest. This general correlation would exist even if there were local variations in the dominant genotypes. However, the incidence of HPV 16 and 18 is similar in all countries; indeed, HPV 16 is slightly higher in the developed nations where cervical cancer is the lowest [17]. The global incidence and mortality of cervical cancer illustrated in Figure 1 does not correlate with the global incidence of HPV 16 and 18.

In 1995 it was known that HPV infection on its own was not sufficient to cause cervical cancer [1,8]. Factors which are known to increase a person’s risk of persistent infection and the progression of lesions to related cancers include [1,2]: a) Multiple partners for the male and female b) Presence of HPV plus other sexually transmitted viruses c) Prostitution [21] d) Sex without a condom/microbicides [13] p.9 e) High parity > 3 children f) Low socioeconomic status: poor hygiene/sanitation/nutrition conducive to sexually transmitted diseases g) Immunosuppression h) Smoking i) Long-term oral contraceptive use and j) older age [22] p.2.

Prostitutes have an increased risk of developing cervical cancer which can be reduced by the use of condoms and microbicides [13,23]. This demonstrates that environmental and lifestyle factors are also necessary for HPV pathogenesis. De Sanjose (2013) states ‘HPV related disease represents a complex mixture of genetics, micro-environment, behaviour and social influences’ [24]. Without these risk factors HPV infections can persist for a lifetime without becoming cancerous [20].

### The efficacy of HPV vaccines

HPV DNA is associated with the development of squamous cell cervical cancer (SCC) and cervical adenocarcinoma (CAC) [25]. In 2003 when the WHO consultation group was investigating the possibility of developing a prophylactic vaccine to prevent these cancers it was decided that a suitable surrogate end-point for the efficacy of the vaccine would be the histologic pre-cursor lesions for these cancers [26]. The histologic pre-cursor lesions are defined as cervical intra-epithelial neoplasia (CIN) grade 2/3 lesions and adenocarcinoma in situ (AIS) [25] p.2. Cervical cancer has a latent period of between 10–30 years between HPV exposure and the development of cervical cancer and this time period for accruing cases was considered unfeasible [26]. The WHO consultation group decided the virological end-point of pre-cancerous lesions in women 15–26 years of age was a useful surrogate for vaccine efficacy studies [13,26]. This was decided even though cervical cancer in this age group is extremely rare and pre-cancerous lesions are common but rarely progress to cancer [13] p.8.

HPV natural history shows that only 5% of HPV infections progress to CIN 2 or 3 within 3 years [11]. Of this 5% many CIN 3 lesions will regress (80%) and approximately 20% progress to invasive carcinoma within 5 years. Of this 20% only 40% progress to invasive carcinoma within 30 years [11].This suggests that the majority of pre-cancerous lesions in young women do not lead to cancer later in life and hence they are not an accurate end-point for determining *how much* cervical cancer can be prevented by an HPV vaccine.

The decision to use this end-point was based on four key features [13,25,27]:

1. They are obligate precursors of cervical cancer.

2. They are closely associated in temporal sequence to the development of invasive cervical cancer.

3. They are associated with a high risk of development of invasive cervical cancer [22] p.1.

4. Reductions in incidence or treatment are shown to result in a reduction in risk of invasive cervical cancer.

The first feature needs qualification. Whilst it is true that pre-cancerous lesions are obligate precursors of cancer, the majority of lesions do not progress to cancer [11,20]. Most high-grade pre-cancerous lesions in young women (90%) regress quickly and without treatment in 2 years [11,13,20]. The incidence of high-grade squamous intraepithelial lesions (HSIL) is highest in this age-group and declines with age [20]. It is stated that cancer is an uncommon outcome of these lesions even in the absence of screening [20] p15. Raffle et al. 2003 observed that at least 80% of HSIL regresses without intervention [20] p 15.

Similarly, features two and three are only true when the environmental and lifestyle risk factors (listed above) are also present [20]. This is demonstrated by the variation in the incidence and mortality rates for cervical cancer between developed and developing countries and between Australia’s indigenous and non-indigenous population. The fourth feature needs to be qualified. In countries where the environmental ‘risk’ factors for pathogenesis have been reduced the majority of HPV infections are not a high ‘risk’ for cervical cancer. Cervical cancer is a rare outcome of all HPV infections with the majority being self-limiting and asymptomatic [1,13,20].

Screening for high-risk HPV infection would identify a very large number of women but only a few of them would be at risk of cervical cancer [20] p 9. This would be the same if all young women are vaccinated – the majority of these women would not be affected by cervical cancer in their lifetime. In addition, there are 15 high-risk HPV subtypes that are implicated in causing cervical cancer and protecting against just 2 does not prevent infection from the other 13 [20]. This is why Merck is now producing a nona-valent vaccine and including 7 more HPV genotypes. Resolved infection from high-risk HPV 16/18 does not protect against other high-risk HPV genotypes [28]. In addition, Gardasil® does not prevent cervical cancer from HPV infection 16 and 18 which was already present at the time of vaccination [25]. In Australia early detection by Pap screening reduced the incidence of cervical cancer by 50% in the decade from 1991 - 2002 [29].

In 2006 when HPV vaccine was licensed and approved for use in the population there was no standard serological assay for detecting HPV antibodies and it was not known what level of antibody titre would be protective against HPV infection [13,30]. An antibody titre against 2 of many oncogenic HPV genotypes (even if a protective level is established) is unable to provide accurate information about the efficacy of HPV vaccines against the burden of cervical cancer. This is because antibody titre is an indication of protection against the infectious agent which in this case is not an *independent* cause of the disease. There is ‘*overwhelming evidence that infection with HPV is necessary, though not sufficient, for development of cancer of the cervix’*[20] p.9.

The expression of disease from an HPV infection depends upon environmental co-factors and most HPV infections are harmless if these co-factors are not also present [1,13,20]. Currently there is no technology to predict which CIN 3 lesions will progress to cancer and which ones will persist for a lifetime without causing disease [11].

### The safety of HPV vaccines

During the two and a half years following the licensure of this vaccine many adverse events to HPV vaccines were reported [31]. Although an analysis of the postlicensure safety surveillance data for the HPV vaccine has been performed, the analysis only included adverse event data from the US [31], despite Gardasil® being licensed in many foreign countries. Slade et al. (2009) also indicate that 68% of the adverse reports for the HPV vaccine in their analysis came from the manufacturer: Merck and Co [31]. Of these reports, almost 89% *did not* provide sufficient identifying information to allow medical review of the individual cases. As a result, the US Centers for Disease Control and Prevention (CDC) vaccine adverse events and reporting system (VAERS) cannot be used to infer causal associations between vaccines and adverse events [32].

This is also because the system is a ‘passive’ system based on voluntary reporting and not an ‘active’ follow up of the health outcomes of all vaccinated individuals [32]. Passive pharmacovigilance systems are not designed to determine causal relationships to adverse reactions or their frequency in the population.

Rare adverse events following immunization (AEFI) were observed more often in the post licensure data than the pre-licensure data [31]. This is a result of:

I. The longer time period over which the data was collected and

II. The larger number of people that were included in the trial.

In the pre-licensure trials adverse events were only actively monitored for 15 days after vaccination [31]. However, in the post-licensure passive surveillance system events were reported that occurred weeks or months after vaccination [31]. It is known that the effects of the chemicals in the vaccines can be latent and appear weeks, months or years after administration [33,34]. The researchers themselves claim that the surveillance system is severely limited [31]. The limitations include:

I. It is a passive system so events are underreported.

II. Not all reported events are systematically validated.

III. Inconsistency in the quality and completeness of reported data.

IV. Reporting biases.

An accurate comparison of adverse events in the clinical trials could not be determined with the unvaccinated group because the placebo was not inert [31]. The manufacturer funded clinical trials used the adjuvant aluminium hydroxyphosphate sulphate, which has been linked to serious adverse events [33], as the placebo in the unvaccinated group [10]. Whilst the WHO states the vaccine is ‘generally safe and well-tolerated’ [13] p.17 this claim does not include a true comparison of vaccinated and unvaccinated females and there has been no *active* follow up of vaccinated individuals.

In the clinical trials 0.1% of women discontinued due to adverse events and 3.6% of pregnant women in both the placebo and vaccinated groups experienced a serious adverse event [13] p. 17. There were 15 to 16 congenital anomalies born in each group [13]. The common factor in these groups was the aluminium adjuvant and it is a signal that there could be many adverse events that have not been causally related to the vaccine because there was no comparison with an inert placebo group in the clinical trials.

The WHO states that background information about the health status of adolescents including acute, chronic and autoimmune diseases should be collected before broad HPV vaccination programs are established [13] p.6. This would ensure that the risks of the vaccine can be properly evaluated. Broad vaccination programs have been rapidly implemented into many countries and the true health effects of this vaccine may never be known if this information has not been collected and if government regulators are using passive surveillance systems.

### Evaluating the cost-effectiveness of HPV vaccines

Government policy-makers in many countries are using epidemiological and economic models to determine the cost-effectiveness of HPV vaccines [35]. There are over 20 different models with considerable variations between them [13,35]. This is due to the significant gaps in the scientific-literature regarding many aspects of HPV natural history and also due to the subjectivity of individual scientists in deciding the level of detail to include in the mathematical models [13,35].

The HPV vaccine is being utilised in many countries even though it is known that there are many uncertainties in the health outcomes predicted by the models because of the use of simplified assumptions [13,15]. Mathematical models depend upon the equations used and the parameter values chosen. Modeling involves many assumptions so good judgment and disciplined integrity by the investigative scientists are vital [36] p 61. Results can be manipulated intentionally or inadvertently so it is important that there is an independent assessment of the models and data used [36].

Almost all HPV models assume that infection, clearance, progression and regression for each HPV type are independent of infection from other types [15]. Although some scientists are now claiming infection from one type influences the chance of infection by another type, more sophisticated multi-type individual based models are needed to properly analyse this possibility [15]. HPV vaccines have been deemed cost-effective for many countries, using mathematical models, even though scientists are claiming that the effects of the vaccine on high-grade lesions and invasive cancer will not be clear for many years [13] p.5;15.

In 2008, Brisson et al. stated that the HPV vaccine trials were showing ‘promising’ results [35] The CE models of HPV prevention in developed countries prior to 2008 concluded that vaccinating girls is ‘likely’ to be cost-effective *if* the duration of vaccine protection is greater than 30 years *or* if booster doses are given when the duration of efficacy is short-term [35]. Other scientists claim that the duration needs to be at least 15 years with 90% efficacy against *at least HPV 16* to be cost-effective [11]. Yet the duration of the vaccine was unknown when the vaccine was marketed to women in 2007 [30] as an effective prevention for cancer [37].

Mathematical models present cost-effectiveness as a ratio (CER) defined as the incremental cost of obtaining a unit of health effect from an intervention when compared to an alternative [35]. Models for HPV vaccine can only produce speculative health outcomes because of the assumptions made about HPV pathogenesis. In the developed countries the majority of HPV infections (90%) are not a high risk for cervical cancer [13]. Empirical evidence of the benefits of the vaccine will not be determined for decades due to the long latent period (10–30 years) between HPV infection and cervical cancer incidence [13] p.5.

The assumptions that have been used in the CER models for HPV vaccines include:

1. HPV DNA on its own is a cause of cervical cancer.

2. HPV 16 and 18 infections are a high risk for developing cervical cancer.

3. High-grade pre-cancerous lesions (CIN 2/3) in 15–26 year old women are a surrogate for cervical cancer.

4. The other 13 + strains of HPV will not infect and progress to cervical cancer.

5. The duration of the vaccine is longer than 10 years.

6. There are few serious side-effects produced by the vaccine.

HPV vaccine is not proven safer or more effective than Pap screening combined with loop electrosurgical excision procedure [11,28] therefore it is important to assess the validity of each assumption regarding pathogenesis and vaccine safety that has been used in the CE models. This knowledge plus the fact that vaccinated women will still need Pap screening must be factored into the assessment. The HPV vaccine costs $Au450 per vaccinated individual (3 doses of vaccine) [38] and this must also be considered against the cost of a Pap test as HPV vaccine does not protect against all oncogenic HPV infections. Pap tests cost approximately $50 which is the cost of the consultation fee with the doctor. In Australia this cost is generally paid by the government and the test is considered to prevent 9 out of 10 cervical cancer cases [38].

## Conclusion

The introduction of the HPV vaccine was based on a number of questionable assumptions that are addressed here.

### HPV DNA is an independent cause of cervical cancer

When scientists trialed this vaccine against pre-cancerous lesions in 2003 it was known that HPV 16 and 18 could persist for a lifetime without causing cervical cancer. Many co-factors had been identified in causality and there were significant gaps in the scientific knowledge regarding the interaction of co-factors with many oncogenic HPV genotypes in pathogenesis. An HPV 16 or 18 infection does not lead to cancer without co-factors being present. The majority of HPV 16/18 infections (90%) are harmless, self-limiting and asymptomatic and are not a high risk of cervical cancer or warts. Environmental and lifestyle factors are known to influence the global incidence and mortality of cervical cancer and this is demonstrated by the lack of correlation between HPV 16 and 18 and the mortality to cervical cancer. HPV 16 and 18 infections are a necessary causal factor in approximately 70% of cases but they are not a sufficient cause.

HPV infection is not an independent cause of cervical cancer and the universal vaccination of all women in developed countries results in the large majority of these women being exposed to the risks of the drug without being at risk of cervical cancer.

### Pre-cancerous lesions in young women as a cervical cancer surrogate

The natural history of HPV 16/18 infection in the 15 – 26 year demographic does not support the conclusion that HPV precancerous lesions are a precursor for cervical cancer: the opposite is true. The majority of pre-cancerous lesions in this demographic regress naturally and do not lead to cancer later in life. This indicates that a measure of efficacy against pre-cancerous lesions (CIN 2 and 3) in young women is an inadequate surrogate for determining *how much* cervical cancer can be prevented with a quadrivalent HPV vaccine.

### HPV genotypes and progression to cervical cancer

It is believed that this vaccine will protect against ~70% of cervical cancer. The assumption is that vaccinated women will not be infected with the 13 other HPV subtypes that are associated with carcinogenesis. Approximately 30% of cervical cancer is linked to HPV genotypes that are not covered in the vaccine. Therefore it is recommended that all vaccinated women should still have regular Pap screening to ensure they are protected. Preventing infection from HPV 16 and 18 assumes that it will prevent *some* cervical cancer but there is no empirical evidence to indicate *how much* cancer it can prevent in developed countries where cervical cancer is already a low risk due to Pap screening programs.

### Duration of the vaccine

The duration of this vaccine was unknown when it was approved by the FDA in 2006 and it is still unknown in 2013. Duration of the vaccine is believed to be at least 5 years as predicted by mathematical modeling performed by the manufacturer. In addition, duration of the vaccine is not an indication of the protection against cervical cancer – only against infection from HPV 16/18. Protection against cervical cancer requires knowledge of the interaction of HPV 16/18 infection with co-factors in pathogenesis as well as the chances of re-exposure to HPV 16/18. In addition, 30% of cervical cancer is not associated with HPV 16 and 18 infections therefore vaccine duration is an incomplete measure of protection from cervical cancer.

### Adverse events

Safety was not adequately investigated in the clinical trials for this vaccine. The trials for this vaccine did not use an inert placebo in the unvaccinated group and they did not study the latent effects of the vaccine components for a year or more after exposure. In addition, there is a lack of knowledge about the harm this vaccine will cause in the population because there is no *active* surveillance system to monitor adverse events. This allows scientists to claim there is no indication that the adverse events reported after HPV vaccination are caused by the vaccine. It is being claimed that these events are a ‘coincidence’ and government regulators are stating the vaccine is ‘safe and effective’ based on a lack of evidence: not evidence-based science.

HPV vaccination programs have been founded on mathematical models that are using uncertain assumptions. HPV vaccines have not been proven to be effective against cervical cancer because inadequate surrogates and end-points have been used to test this hypothesis and cervical cancer takes 10–30 years to develop. Approximately 90% of HPV infections clear spontaneously and are asymptomatic and harmless: only a fraction lead to cervical cancer over 2–3 decades. This is a significant factor in the broad use of an HPV vaccine in adolescents. Vaccination programs are targeting 11–12 year olds in which the risk of cancer death is zero. In comparison, the risk of vaccine injury or death is very real. This risk may be small or large but it is necessary to have an accurate estimate before broad vaccination programs are implemented. Governments that implement HPV vaccination programs are shifting the risk and not eliminating the risk of adverse health outcomes.

In addition, vaccination programs are very expensive compared to the cost of screening programs. Pap screening is almost 100% effective in preventing cervical cancer and virtually risk free. In contrast, the HPV vaccine is very expensive and it cannot prevent 30% of cervical cancer: Pap screening will still be required. HPV vaccines will not be cost-effective against screening programs until they can prevent 100% of cervical cancer without significant adverse events.

Currently the benefit of the vaccine against the burden of cervical cancer in developed countries is unknown and there are risks of injury and death that have not been accurately determined. HPV vaccines are not demonstrated to be safer or more effective than Pap screening combined with surgical procedures. Hence it follows that implementing broad HPV vaccination programs is not cost-effective in countries where regular Pap screening programs are available and will still be required. HPV vaccines in vaccination programs in these countries are offering uncertain benefits in reducing the burden of cervical cancer and may cause more harm than good due to the lack of investigation of their long-term safety.

## Abbreviations

HPV: Human papillomavirus; SCC: Squamous cell cervical cancer; CAC: Cervical adenocarcinoma; AIS: Adenocarcinoma *in situ*; HSIL: High-grade squamous intraepithelial lesions; WHO: World Health Organisation; IARC: International Agency for Research on Cancer; PCR: Polymerase chain reaction; US: United States; CDC: Centre for disease control and prevention; VAERS: Vaccine adverse event reporting system; CE: Cost-effectiveness; CER: Cost-effectiveness ratio.

## Competing interests

The authors declare that she has no competing interest.
